# A Rare Case of Pregnancy After Surgical Treatment for Stage III Hidradenitis Suppurativa

**DOI:** 10.7759/cureus.45370

**Published:** 2023-09-16

**Authors:** Sanjana D Nalla, Sowjanya Kurakula, Maheshwari Nallur Siddaraju

**Affiliations:** 1 Medical School, Lake Erie College of Osteopathic Medicine, Erie, USA; 2 Obstetrics and Gynecology, Lifeline Medical Associates, Edison, USA; 3 Family Medicine, Medical Offices of Dr. Shaswathi Kale, San Jose, USA

**Keywords:** multidisciplinary care, sex hormones, depression, comorbidities, remission, surgical management, pregnancy, chronic inflammatory skin disease, hidradenitis suppurativa

## Abstract

Hidradenitis suppurativa is a chronic inflammatory skin condition primarily affecting areas with apocrine glands. It commonly manifests as painful nodules, abscesses, and sinus tracts, significantly impacting the patient's well-being. In this report, we discuss a case of a 32-year-old pregnant woman with Hurley stage III hidradenitis suppurativa who had undergone surgical reconstruction of the vulva before conception. The patient underwent a cesarian section and received topical treatment for her lesions near the genitalia. The importance of early identification, a collaborative approach involving multiple specialists, and individualized treatment strategies for managing this debilitating condition during pregnancy are emphasized in this case study.

## Introduction

Hidradenitis suppurativa, also known as acne inversa, is a chronic inflammatory skin disease characterized by painful subcutaneous nodules and abscesses. This condition primarily affects areas of the body that are rich in apocrine sweat glands, such as the perineal, gluteal, inguinal, and axillary regions [1]. The Hurley classification system is used to identify the progression of the disease with stage I showing little to no scarring to stage III where lesions eventually progress into more extensive scarring and formation of sinus tracts throughout the body, greatly impacting quality of life [2]. While the etiology of hidradenitis suppurativa is elusive, research has shown that it could be due to an amalgam of immune system dysregulation, genetics, and lifestyle factors such as cigarette smoking, obesity, and stress [3].

The prevalence of hidradenitis suppurativa in females is markedly higher than in males with the average age of onset being 22.1 years [4]. Due to the female predominance, hidradenitis suppurativa not only has a significant impact on the patient’s physical and mental well-being but also plays a large role and is associated with many comorbidities that can be deleterious during pregnancy. Pregnant patients with hidradenitis suppurativa have been found to have an increased risk of complications including spontaneous abortion, gestational diabetes, gestational hypertension, preeclampsia, and preterm birth [5]. Utilizing current knowledge of this unpredictable condition to effectively diagnose and provide treatment options early on is crucial in the management of this condition, especially in pregnancy and postpartum.

## Case presentation

A 32-year-old female patient presented to the antenatal clinic in her first trimester with a past surgical history of multiple skin debridement and graft procedures for hidradenitis suppurativa lesions on the groin and vulva over a few years. The patient first experienced encounters of hot boils in the axilla that would burst open during her early twenties which were successfully treated topically. Her menstrual cycles were regular, and painful, with adequate flow. She was normotensive and her blood sugar levels were within normal range throughout her pregnancy. The patient had no significant past medical history and did not consume alcohol or take any illicit drugs. The patient had no known allergies. Family history was significant for hypertension and diabetes. The review of systems was unremarkable. In the first antenatal visit, on examination, the patient’s BMI was 33.4, urine pregnancy test was positive at 5 weeks. Her vital data were within normal limits. No abnormal findings were detected on general and abdominal examination.

She had a spontaneous vaginal delivery in 2014, during the immediate postpartum she was diagnosed for the first time with hidradenitis suppurativa lesions in the axilla region. She described her pain as excruciating at a rate of 10 on the pain scale with a 0 score being a painless state. As she was breastfeeding, her antibiotics were chosen cautiously as per her swab culture and sensitivity results from the lesions due to concerns for drug safety. The patient was advised to use warm compressions and oral antibiotics which did not relieve her pain. She had on-and-off painful lesions occasionally during the following years.

In 2020, the disease flared up once again causing her to notice a small bump on her foot, prompting her to return to the clinic. The patient was started on a tumor necrosis factor inhibitor, Humira 20 mg/0.4 mL shots weekly; however, the treatment aggravated her hidradenitis suppurativa causing increased irritation and itching in the groin region. She developed painful multiple abscesses with draining sinuses in the areas including the groin, upper medial thighs, vulva, and buttock regions. Throughout this time the patient experienced multiple crying episodes and depression due to the intense pain. After Humira was not proven to be effective in the management of the patient’s hidradenitis suppurativa, surgical management was recommended as the patient had multiple skin sinuses that had spread to her vulva and buttocks regions.

In July 2020, the patient was scheduled for her first of three total skin debridement surgeries by a plastic surgeon. During this surgery, debridement and reconstruction of the patient’s mons pubis as well as the left and right medial thighs and buttocks were completed. The patient repeated this surgery in September and December 2020 with a focus on the buttock, medial thigh, axillary, lower back, and mons pubis regions. In between surgeries, the patient discontinued her previous treatments for her hidradenitis suppurativa and received approximately 15 hyperbaric oxygen therapy sessions; however, she stated that the efficacy of this treatment was minute. A final skin graft surgery was scheduled in January 2021 where portions of skin were extracted from the patient’s thighs. The patient experienced discharge, itching, and draining of pus from the lesions during the entirety of her surgical treatment. She was placed on a few drains and was closely examined with multiple follow-ups after each surgery. A wound vac was utilized to remove the pus and further the healing process. The patient had a pain-free life after undergoing surgical treatment.

Once the patient conceived in 2022 with her second child, she was placed on oral dicloxacillin, and metronidazole 0.75% vaginal gel for her minor complaints such as scanty pus discharge from occasional draining sinuses on a few occasions. During the pregnancy, there were no significant issues that arose for the patient and she did not experience any flare-ups with her hidradenitis suppurativa.

In the third trimester patient consulted with a plastic surgeon to seek an opinion on the mode of delivery. The patient was examined at 36 weeks which revealed extensive scarring of the perineum and contracted skin of the introitus. Given her multiple surgical reconstructions near her vulva, groin, and medial thigh regions, a lower segment cesarean section was advised. Transverse incision over the skin was given at a higher level to avoid the scarred tissue region from previous vulval reconstruction.

**Figure 1:**
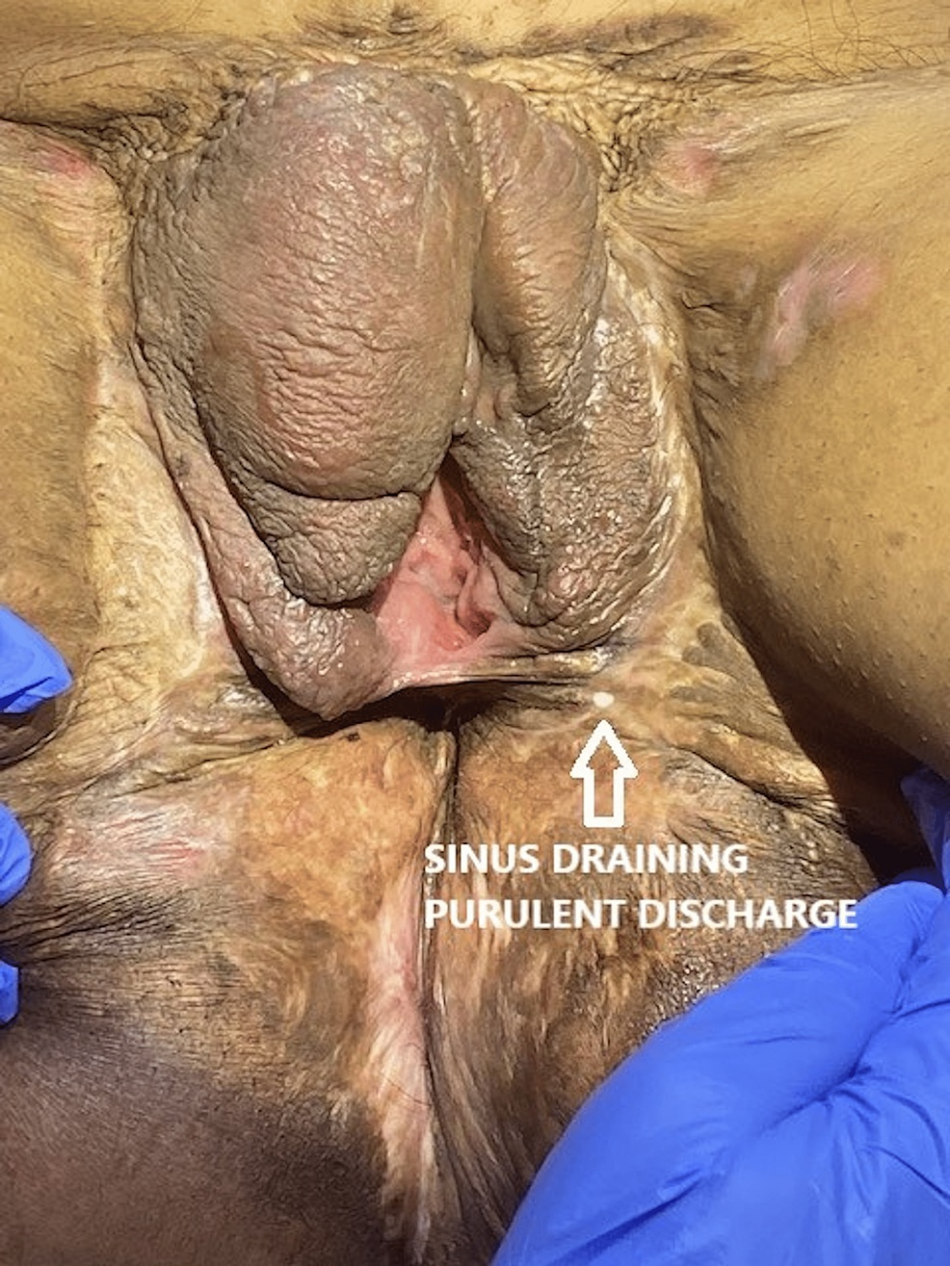
Extensive scarring and contracture involving the vulva, perineum, and groin. A sinus opening with pus discharge is seen below the introitus

**Figure 2:**
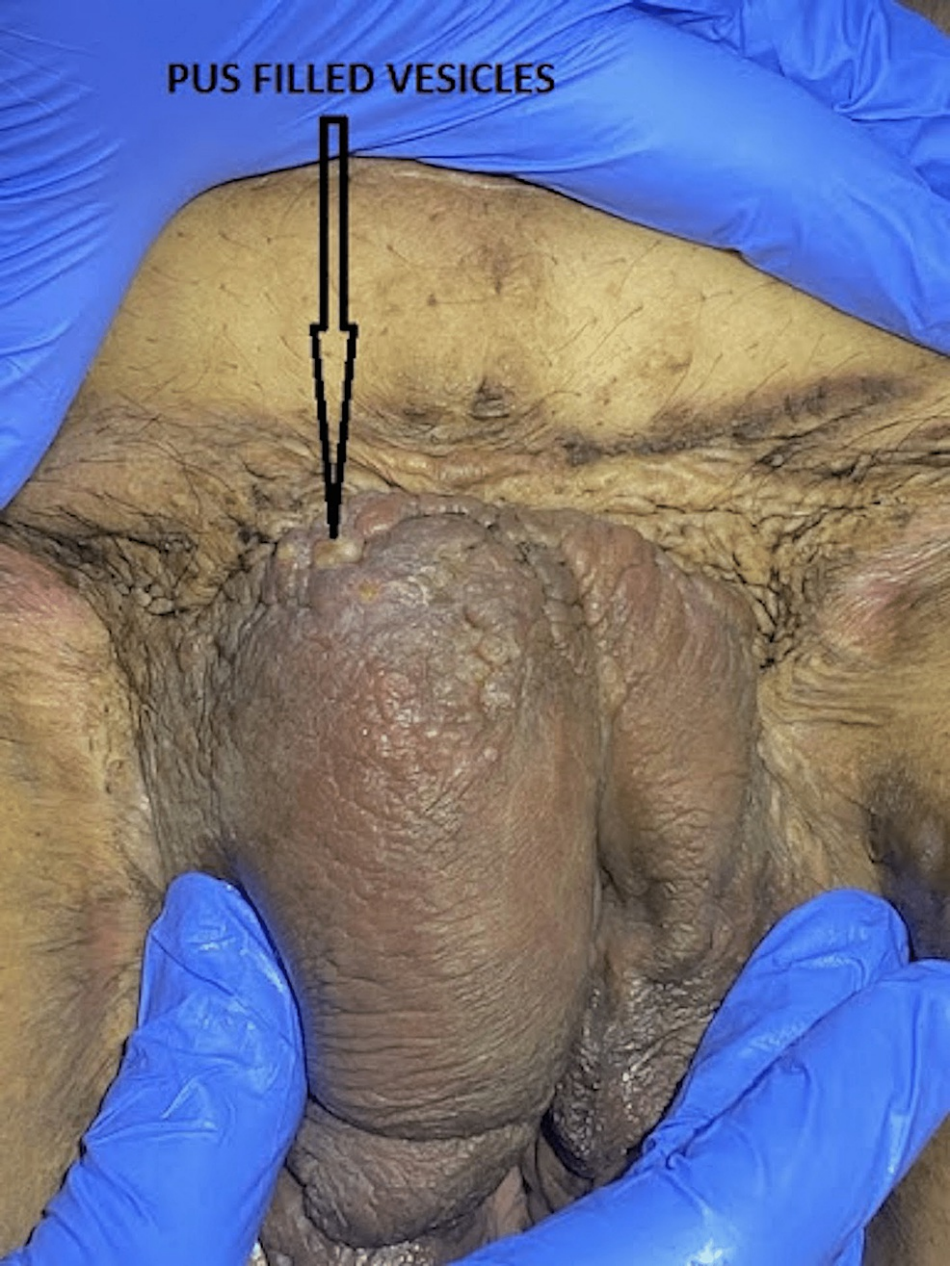
Multiple grouped pustules are seen on the pubis. Disfigured pubis due to scarring can be seen

**Figure 3:**
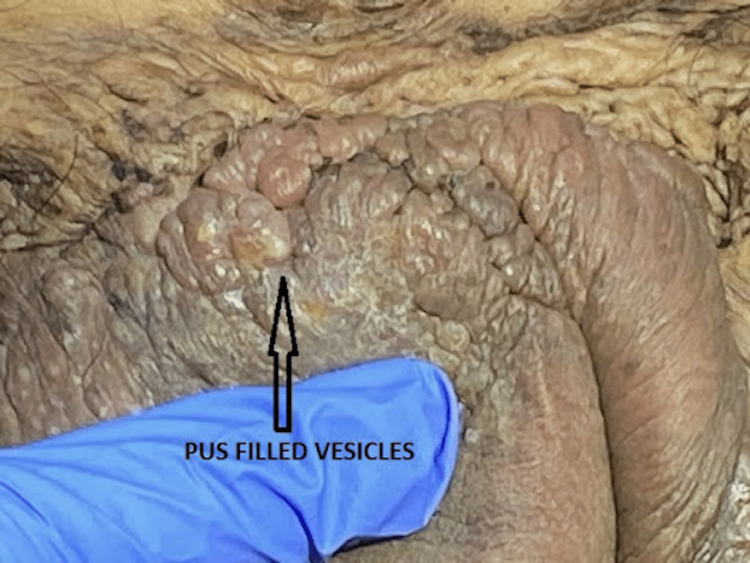
Multiple grouped pustules are seen on the pubis

**Figure 4:**
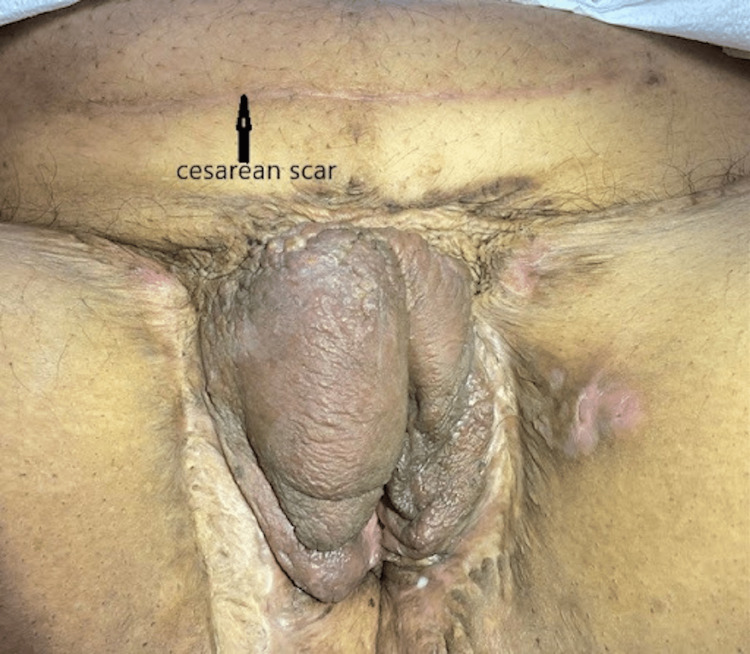
Healed high transverse cesarian scar at 6 weeks postpartum

During her 6th week postpartum visit, the patient was in good health with a few draining lesions in the perineum [Figure 1-4].

Her medications include ferrous fumarate 89 mg, and vitamin D3 2,000 units. During the postpartum period and until the present day the patient’s hidradenitis suppurativa did not exacerbate and she is currently on no treatment for her condition.

## Discussion

In the United States, the standardized point prevalence of the condition is roughly 2.4 times higher in women compared to men. Additionally, among patients, the prevalence is approximately three times higher in Black individuals compared to White individuals. The progression of the disease can be categorized using the Hurley classification system. Stage I indicates the absence of tunnels or scarring, stage II indicates the presence of tunnels or scarring mixed with areas of unaffected skin, and stage III indicates widespread tunnels or scarring that replaces most if not all, of the normal skin in a specific anatomical region [2].

Hidradenitis suppurativa primarily affects women in the age group of 18 to 39 years [5]. The average age of onset for hidradenitis suppurativa is 22.1 years, and it typically persists for approximately 19 years. However, the course of the disease can vary widely [4]. Tobacco smoking is considered an important predisposing risk factor. Hidradenitis suppurativa is associated with several comorbidities that can lead to negative outcomes for both the mother and the fetus. These comorbidities include pre-pregnancy obesity, diabetes mellitus (DM), polycystic ovary syndrome (PCOS), hypertension (HTN), thyroid disease, alcohol use disorder, and substance use disorder [5]. Obesity has a significant impact on hidradenitis suppurativa due to the mechanical shearing effect over intertriginous areas, promoting inflammation along with added insulin resistance [6].

Psychiatric comorbidities are prevalent and significant in hidradenitis suppurativa, with depression being one of the most common and severe [2,6]. The estimated prevalence of depression in hidradenitis suppurativa patients can reach as high as 43%, and there have been reports of suicidal ideation or attempts in 12% of patients. Tragically, individuals with hidradenitis suppurativa, especially women, have higher rates of completed suicide [2].

The follicular pyodermas, including folliculitis, furuncles, and carbuncles, are among the most frequently encountered differential diagnoses for hidradenitis suppurativa. There is a notable correlation between sex hormones and hidradenitis suppurativa/autoimmune diseases. The higher prevalence among females suggests that women may have a heightened sensitivity to androgens, highlighting the connection between sex hormones and the development of hidradenitis suppurativa. Hidradenitis suppurativa may experience remission or partial remission during pregnancy and breastfeeding. Generally, it is considered a benign, mild, and chronic condition with intermittent pain. It exhibits acute exacerbations, and premenstrual flares, and can resolve after menopause. Remission periods can range from weeks to months, while flares may be continuous or intermittent. Additionally, exposure to androgenic progestins, such as medroxyprogesterone acetate (MPA) or levonorgestrel, can also trigger or exacerbate hidradenitis suppurativa symptoms [4].

In a study that examined pregnancy outcomes, 1,600 pregnant women with hidradenitis suppurativa were compared to 53,278 pregnant women without hidradenitis suppurativa. The results showed that among women with hidradenitis suppurativa, the rate of live births was 82%, spontaneous abortion occurred in 15.5% of cases, stillbirth was reported in 0.5% of cases, and preterm birth affected 9.1% of pregnancies. Among the 1,600 pregnant women, the following percentages were observed: 11.6% developed gestational diabetes mellitus (DM), 6.1% developed gestational hypertension (HTN), 6.6% developed preeclampsia, and 32.4% underwent a cesarean section [5]. 

A study summarised that 127 patients with hidradenitis suppurativa during pregnancy posed no risk of poorer pregnancy and neonatal outcomes [7]. A study on women with hidradenitis suppurativa in pregnancy reported that 3.1% of patients with anogenital hidradenitis suppurativa who delivered vaginally described that their condition interfered with delivery, and 23.5% revealed that delivery caused flare-ups. Among patients who underwent a cesarean section delivery (44.2%), 33.9% reported impaired incision healing secondary to hidradenitis suppurativa, and 51.2% reported developing new hidradenitis suppurativa inflammatory nodules within the incision site [8]. A study concluded that about 25% of women with hidradenitis suppurativa during pregnancy experienced improvement of symptoms, whereas the majority had either stable disease or worsening of symptoms. Around 60% had flare-ups of hidradenitis suppurativa lesions during the postpartum period [9].

Our case who underwent extensive reconstructive surgeries on the vulva and gluteal regions has never reported flare-ups of lesions during pregnancy or after cesarean delivery except for a few occasions of painless draining sinuses with scanty purulent discharge.

According to a study, a woman at 26 weeks of pregnancy with worsening hidradenitis suppurativa lesions on her breast underwent debridement and excision followed by primary wound closure after 1 week in the late second trimester successfully breastfed her newborn in postpartum. They reported effective surgical management involving the excision of the entire lesion with areas of fibrosis, sinuses, and fistulae [10].

Hidradenitis suppurativa can be accompanied by various complications, including the development of anal and perianal fistulas as well as arthropathy. Additionally, some individuals may experience significant genital swelling, and metabolic issues such as anemia and hypoproteinemia, and in rare cases, amyloidosis may occur. Scarring in the axillae and groin regions of hidradenitis suppurativa can lead to the formation of strictures in the anal, urethral, and rectal areas. Currently, there is no single treatment or cure that is universally effective for hidradenitis suppurativa/autoimmune diseases. The only reported permanent cure has been observed in cases of severe hidradenitis suppurativa (Hurley stage III) through extensive surgery. Managing hidradenitis suppurativa requires a combination of metabolic, medical, and surgical approaches, as well as lifelong gentle and non-traumatic care for patients [4].

Early diagnosis and optimal treatment of hidradenitis suppurativa by obstetricians and gynecologists can effectively decrease morbidity associated with the condition and potentially improve the overall disease progression [2]. Our patient developed episodes of depression on several occasions due to her constant and severe painful lesions especially when she did not respond to her medical management and during aggravated disease triggered after adalimumab therapy. 

Several conservative treatment options exist for hidradenitis suppurativa women including antibiotics, biologics, metformin, immunosuppressants, hormonal therapy, retinoids, laser therapy, cryotherapy, intralesional steroids, smoking cessation, weight reduction, stress reduction, hydrotherapy, zinc therapy, analgesics. Currently, there is a dire need for research due to limited data available on the safety and efficacy of various treatment modalities of hidradenitis suppurativa during pregnancy [6].

## Conclusions

This case highlights the challenging nature of hidradenitis suppurativa, primarily affecting women. Despite unsuccessful treatments, including Humira, surgical management was required. After multiple debridement surgeries and skin grafting, the patient experienced pain relief. The association of hidradenitis suppurativa with comorbidities impacting pregnancy outcomes emphasizes the need for careful management during pregnancy. Depression's prevalence in hidradenitis suppurativa patients and the potential role of sex hormones in hidradenitis suppurativa development require further research for better treatment approaches. A comprehensive care approach involves multidisciplinary collaboration involving primary care physicians, obstetricians, dermatologists, and psychiatrists, and personalized treatment plans are crucial for managing severe hidradenitis suppurativa, particularly during pregnancy. Further research is essential to enhance hidradenitis suppurativa management and patients' quality of life.
